# Preferences for breast cancer prevention among women with a *BRCA1* or *BRCA2* mutation

**DOI:** 10.1186/s13053-020-00152-z

**Published:** 2020-09-29

**Authors:** Carol A. Mansfield, Kelly A. Metcalfe, Carrie Snyder, Geoffrey J. Lindeman, Joshua Posner, Sue Friedman, Kathleen Cuningham Foundation Consortium for Research into Familial Breast Cancer, Henry T. Lynch, Steven A. Narod, D. Gareth Evans, Alexander Liede

**Affiliations:** 1 RTI Health Solutions, Research Triangle Park, 3040 Cornwallis Road, PO Box 12194, Durham, NC 27709-12194 USA; 2 Women’s College Hospital, University of Toronto, Toronto, Canada; 3 Creighton University, Omaha, NE USA; 4 CHI Health Creighton University Medical Center, Omaha, NE USA; 5 The Royal Melbourne Hospital, Parkville, Australia; 6 Peter MacCallum Cancer Centre, Melbourne, VIC Australia; 7 The Walter & Eliza Hall Institute of Medical Research, Parkville, VIC Australia; 8 The University of Melbourne, Parkville, VIC Australia; 9 Facing Our Risk of Cancer Empowered (FORCE) Advocacy Organization, Tampa, Florida USA; 10 Kathleen Cuningham Foundation Consortium for Research into Familial Breast Cancer (kConFab), Research Department, Peter MacCallum Cancer Centre, Melbourne, Australia; 11 Manchester Centre for Genomic Medicine, MAHSC, Division of Evolution and Genomic Sciences, University of Manchester, Manchester, UK; 12 AbbVie Inc, Dublin, Ireland

**Keywords:** *BRCA1*, *BRCA2*, High-risk women, Risk-reducing surgeries, Prevention, Survey, Preferences, Choices, Unaffected women, International study

## Abstract

**Background:**

Women with a *BRCA1* or *BRCA2* mutation have high lifetime risks of developing breast and ovarian cancer. The decision to embark on risk reduction strategies is a difficult and personal one. We surveyed an international group of women with *BRCA* mutations and measured choices and sequence of breast cancer risk reduction strategies.

**Methods:**

Women with a *BRCA1/2* mutation and no previous cancer diagnosis were recruited from the US, Canada, the UK, Australia, and from a national advocacy group. Using an online survey, we asked about cancer-risk reduction preferences including for one of two hypothetical medicines, randomly assigned, and women’s recommendations for a hypothetical woman (Susan, either a 25- or 36-year-old). Sunburst diagrams were generated to illustrate hierarchy of choices.

**Results:**

Among 598 respondents, mean age was 40.9 years (range 25–55 years). Timing of the survey was 4.8 years (mean) after learning their positive test result and 33% had risk-reducing bilateral salpingo-oophorectomy (RRBSO) and bilateral mastectomy (RRBM), while 19% had RRBSO only and 16% had RRBM only. Although 30% said they would take a hypothetical medicine, 6% reported taking a medicine resembling tamoxifen. Respondents were 1.5 times more likely to select a hypothetical medicine for risk reduction when Susan was 25 than when Susan was 36. Women assigned to 36-year-old Susan were more likely to choose a medicine if they had a family member diagnosed with breast cancer and personal experience taking tamoxifen.

**Conclusions:**

Women revealed a willingness to undergo surgeries to achieve largest reduction in breast cancer risk, although this would not be recommended for a younger woman in her 20s. The goal of achieving the highest degree of cancer risk reduction is the primary driver for women with *BRCA1* or *BRCA2* mutations in selecting an intervention and a sequence of interventions, regardless of whether it is non-surgical or surgical.

## Background

Women with inherited mutations in the *BRCA1* or *BRCA2* genes have a greatly increased risk of developing early onset breast cancer and ovarian cancer and face difficult decisions about options for risk reduction and early detection [1–5]. Risk-reducing bilateral mastectomy (RRBM) and risk-reducing bilateral salpingo oophorectomy (RRBSO) surgeries, and routine surveillance with mammography or magnetic resonance imaging, are among the options often considered. Few non-surgical interventions are available to reduce breast cancer risk in these high-risk women, and only a very small percentage of women with *BRCA* mutations take chemoprevention options such as tamoxifen, other selective estrogen receptor modulators, or aromatase inhibitors.

The decision to embark on elective risk-reducing surgery is a very complicated and personal one. Angelina Jolie, the American actress and humanitarian, shared her own personal patient journey after receiving a diagnosis of *BRCA* mutation for the benefit of other similarly-diagnosed women in an opinion piece in the *New York Times* [6]. Prophylactic surgery reduces the risk of cancer and the stress associated with that risk, but the surgery itself is not without risk and can have a significant psychological impact on the women who undergo such procedures [7].

Preclinical studies evaluating the receptor activated nuclear factor kappa-B ligand (RANKL) on breast cell proliferation, have shown that RANKL driven progesterone signaling can play a critical role in breast cancer tumorigenesis among *BRCA1* mutation carriers [8–14]. Consequently, clinical trials using the RANKL inhibitor, denosumab [15], have been initiated as a possible chemoprevention alternative for women with a *BRCA1* mutation [16, 17].

This research raises the question of how a new drug treatment option would fit in among the existing alternatives, and what factors might shape a woman’s choice to include such a therapy. To better understand how a new option might change real-world treatment patterns, this study was designed to identify ways in which women’s prophylactic treatment choices might vary with the addition of a new non-surgical option. To address this objective, women with a *BRCA1* or *BRCA2* mutation, and with no personal history of cancer, were asked if they would choose their original treatment option (if they had the choice to make again) or other options, including a hypothetical chemoprevention option. Systematic variation in women’s choices—as a function of the woman’s individual characteristics—were explored. In addition, we compared respondents’ choices for themselves to the respondent’s choices for a hypothetical woman of varying age who has just learned she has *BRCA1* or *BRCA2* mutation.

## Methods

### Survey instrument

**Fig. 1:**
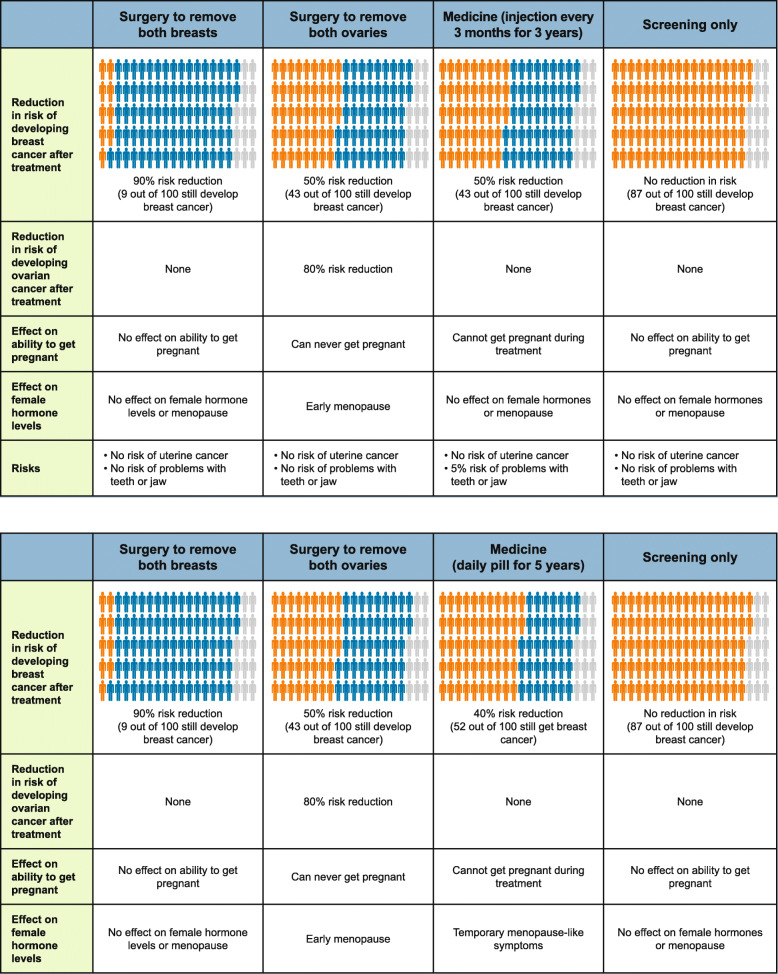
Treatment Choices With Denosumab-Like and a Tamoxifen-Like Medicines. a. Medicine 1, a Denosumab-Like Medicine^a^. b. Medicine 2, a Tamoxifen-Like Medicine^a^. ^a^ Illustration describes the choices used to elicit women’s preferences for breast cancer risk reduction

The survey instrument included multiple questions to assess preferences for risk-reducing treatment options. As part of the survey, respondents were presented with four potential treatment options (Fig. 1a and b). The four treatments in Fig. 1a and b have features that correspond to RRBM, RRBSO, a hypothetical medicine with characteristics like denosumab (Fig. 1a) or tamoxifen (Fig. 1b, respondents were randomly assigned to one medicine or the other), and screening only. Respondents first read through descriptions of the surgeries, medicines, and the screening-only option before considering how they would choose to sequence these therapeutic options for their own care.

Respondents were asked to think back to when they first found out about their *BRCA1* or *BRCA2* mutation and to assume that the 4 choices in the table were all available. They were then asked to select the first action they would take if the four options presented were their only choices. The survey directed respondents who selected screening only, to indicate how long they would screen before moving on to another treatment. Respondents were asked to continue reporting their treatment choices in sequence until they reached the point at which they would choose not to take any additional action, and to continue only with screening. The percentage of risk reduction associated with each treatment stayed fixed, which potentially overstates the risk reduction for the treatments that are not selected as the first treatment, which we discuss in the Limitations section.

After answering the series of questions about how she would choose to sequence her own treatments, respondents were asked to make the same choices for Susan, a hypothetical woman described as follows:

Susan is a (25/36)-year-old woman who has no children, but she plans to have children in the next 5 years. One month ago, Susan found out that she has a mutation in the BRCA1 gene. Based on her family history and the type of mutation that was identified, her genetic counselor told her that she has an 87% chance of developing breast cancer before she turns 70.

For this portion of the survey, women randomly assigned to Medicine 1 were asked to select choices for Susan when she was age 25 years and women assigned to Medicine 2 were asked to select choices for Susan when she was age 36.

The survey questions and descriptive text were written in patient-friendly language and the survey was pretested in 14 one-on-one interviews to improve comprehension and wording. In addition to questions on treatment preferences, the survey also collected information on the respondent’s actual treatment history. The treatment history included questions on RRBM, RRBSO, and whether they had taken a prescription medication such as tamoxifen, raloxifene, or an aromatase inhibitor to reduce their risk of developing breast cancer. The methods and results of a discreet-choice experiment quantifying women’s preferences for breast cancer risk-reduction strategies included in the survey have been described in detail elsewhere [18]. The survey also included questions on psychological stress (the results from these questions are described elsewhere [7]), family history of breast and ovarian cancer, and demographic questions.

### Study sample and recruitment

Women aged 25 to 55 years with an inherited mutation in the *BRCA1* and/or *BRCA2* gene who were unaffected with breast or ovarian cancer were eligible to participate in the online survey. Respondents were recruited through Facing Our Risk of Cancer Empowered (FORCE), a patient advocacy group, and through the research registries at Creighton University (United States), Women’s College Hospital (Canada), Royal Melbourne Hospital (Australia), Kathleen Cuningham Foundation Consortium for Research into Familial Breast Cancer at the Peter MacCallum Cancer Centre (Australia), and Manchester Centre for Genomic Medicine (United Kingdom). FORCE recruited respondents through its website, newsletters, and social media. Respondents recruited through FORCE provided a self-reported diagnosis of their *BRCA1* or *BRCA2* status. The clinical sites identified respondents on their registries who met the study inclusion criteria. Potential respondents were mailed invitation letters with the URL of the online survey and a unique password and provided informed consent prior to their inclusion in the study. Institutional review boards at RTI International and all participating sites approved the study.

### Statistical analysis

The data on the women’s stated sequence of treatment choices for themselves and the hypothetical Susan were summarized with descriptive statistics and using a Sunburst diagram, which displays hierarchical data in a series of concentric circles.

For the full sample, as well as separately for women who were assigned to the two different medicine profiles, multivariable logistic regression models were used to predict the likelihood that a woman in the study would choose to take a hypothetical chemoprevention medication as a function of the woman’s characteristics. Additional multivariable logit models were used to predict the likelihood that respondents would select a chemoprevention medication for the hypothetical woman (Susan) as a function of the respondent’s characteristics and Susan’s age (25 years old, or 36 years old).

The respondents’ actual choices for risk-reducing treatments since the time of their diagnosis with the *BRCA1* and/or *BRCA2* gene were summarized for comparison. Women who had not had surgery themselves were asked how likely they were to get a RRBM or a RRBSO in the future, with the response captured on a 4-point Likert scale that ranged from “very unlikely” to “very likely”, and data for these categorical responses were also summarized. The multivariable logistic regression models were generated using SAS software, version 9.4 (SAS Institute Inc., Cary, North Carolina). Summary statistics and associated *P*-values were generated using Stata, version 16 software (StataCorp, College Station, Texas). All P-values < 0.05 (two-tailed) were considered statistically significant.

## Results

Between January 2015 and March 2016, the clinical sites mailed 1174 letters to potentially eligible women, 383 women accessed the survey, and 338 women met the inclusion criteria. Through FORCE, 1374 women accessed the online survey, and 494 met the inclusion criteria. Of the 832 women from FORCE and the clinics who met the eligibility criteria, 598 respondents answered at least one of the treatment sequencing questions and were included in the analysis. Overall, the average age of the respondents was approximately 41 years, and the average time since the women learned about their gene mutation was 4.8 years. Approximately 52% of the respondents reported a BRCA1 mutation, 46% reported a BRCA2 mutation, and 1% reported both BRCA1 and BRCA2 mutations (Table 1).

**Table 1:** Respondent Characteristics, *N* = 598 (unless otherwise noted)

Characteristic	Full Sample (***N*** = 598)
**All respondents**	
Age, mean (SD), years	40.9 (8.2)
Age 40 years or older	335 (56%)
Has children	416 (70%)
Hopes to have children in future (after mutation identified) or undecided^a^	268 (45%)
White or Caucasian	558 (93%)
Married/living as married/civil partnership	440 (74%)
Higher education (defined as post-secondary/any college and higher)	401 (67%)
Employed full time	329 (55%)
Country of residence	
United States	331 (55%)
United Kingdom	117 (20%)
Australia	119 (20%)
Canada	31 (5%)
Mutation	
*BRCA1*	310 (52%)
*BRCA2*	273 (46%)
*BRCA1* and *BRCA2*	8 (1%)
Don’t know or not sure	7 (1%)
Mean time since genetic test, years (SD)	4.8 (4.3)
First degree relative with breast cancer	374 (63%)
First degree relative with ovarian cancer	122 (20%)
Risk-reducing treatments	
RRBM only	95 (16%)
RRBSO only	112 (19%)
RRBM and RRBSO	198 (33%)
Has taken a prescription medication, such as tamoxifen, raloxifene, or an aromatase inhibitor [anastrazole, exemestane]	33 (6%)
**Among women who did not report RRBM surgery**	
n	305
Very likely or somewhat likely to undergo RRBM in the future^b^	193 (63%)
**Among women who did not report RRBSO surgery**	
n	288
Very likely or somewhat likely to undergo RRBSO in the future^c^	255 (89%)
**All respondents**	
n^d^	587
Assigned to Susan, age 25 years	285 (48%)
Assigned to Susan, age 36 years	302 (51%)

*RRBM* Risk-reducing bilateral mastectomy, *RRBSO* Bilateral salpingo oophorectomy, *SD* Standard deviation

^a^ Includes women who indicated that they wanted to have children or have more children in the future or were undecided about having children or having more children and women who have children that were born after their *BRCA 1* or *BRCA2* mutation was identified

^b^ Based on the question “How likely are you to have a surgery to remove both breasts (risk-reducing bilateral mastectomy) in the future?” with the response choices very likely, somewhat likely, unlikely, very unlikely, don’t know or not sure

^c^ Based on the question “How likely are you to get your ovaries and fallopian tubes removed in the future?” with the response choices very likely, somewhat likely, unlikely, very unlikely, don’t know or not sure

^d^Three respondents did not complete the full survey, thus were not assigned to a Susan age group and did not answer any of the four Susan treatment sequencing questions. An additional four respondents assigned to Susan, age 25 and four respondents assigned to Susan, age 36 also did not answer any of the four Susan treatment sequencing questions presented in the survey. Thus, these 11 respondents are not included in the two Susan group sample totals summarized in Table 1 and were not included in the Susan treatment sequence analysis presented in this article

Table 1 reports the percentages of women who reported getting each of the treatments. Thirty-three percent had both an RRBM and a RRBSO, while 19% had a RRBSO only and 16% had an RRBM only. Six percent reported taking a prescription medicine such as tamoxifen, raloxifene, or an aromatase inhibitor.

Table 2 reports the percentage of time that each of the treatment options in Figs. 1a and b was selected. The majority of respondents indicated that, if they had the decision to make again, they would have pursued surgery: 78.6% indicated they would get an RRBSO as part of their risk-reduction strategy, and 73.7% indicated they would get an RRBM. Almost 30% said they would take a hypothetical medicine as part of their treatment sequence; 31.1% said they would take Medicine 1 (the medicine with features like denosumab), while 28.5% said they would take Medicine 2 (the medicine with features like tamoxifen).

**Table 2:** Responses to Treatment Sequencing Questions (*N* = 598, unless otherwise noted)

Summaries	Treatment Option	Value, n (%)
**Respondent’s treatment selection for themselves**^**a**^		
Treatment option ever selected as part of treatment sequence	RRBM	441 (73.7)
	RRBSO	470 (78.6)
	Medicine (Medicine 1 or Medicine 2)	178 (29.8)
Among respondents assigned to Medicine 1^b^		
	n	289
	Medicine 1	90 (31.1)
Among respondents assigned to Medicine 2^b^		
	n	309
	Medicine 2	88 (28.5)
**Treatment advice for Susan, age 25 years or age 36 years**^**a**^		
Among respondents assigned to Susan, age 25 years^c^		
Treatment option ever selected as part of the Susan treatment sequence	n^d^	285
	RRBM	157 (55.1)
	RRBSO	110 (38.6)
	Medicine 1	87 (30.5)
Among respondents assigned to Susan, age 36 years^c^		
Treatment option ever selected as part of the Susan treatment sequence	n^e^	302
	RRBM	214 (70.9)
	RRBSO	147 (48.7)
	Medicine 2	68 (22.5)

*ONJ* Osteonecrosis of the jaw, *RRBM* Risk-reducing bilateral mastectomy, *RRBSO* Bilateral salpingo oophorectomy

^a^Respondents were asked to think back to when they first discovered their *BRCA1* or *BRCA2* mutation and to assume that the 4 choices presented in Fig. 1a/1b were all available. They were then asked to select the first action they would take if the four options presented were their only choices. The survey directed respondents who selected screening only to indicate how long they would screen before moving on to another treatment. Respondents were asked to continue reporting their treatment choices in sequence until they reached the point at which they would choose not to take any additional action, and to continue only with screening. Respondents were then asked to make the same choices for Susan, age 25 or Susan, age 36; a hypothetical woman described on the Methods section

^b^Respondents were randomly assigned to either Medicine 1 (50% breast cancer risk reduction, take for 3 years, cannot get pregnant during treatment, no effect on female hormone levels, 5% risk of ONJ, no risk of uterine cancer, injection at doctor’s office every 3 months) or Medicine 2 (40% breast cancer risk reduction, take for 5 years, cannot get pregnant during treatment, temporary menopause-like symptoms, no risk of ONJ, 1% risk of uterine cancer, daily pill)

^c^All respondents assigned to Susan, age 25 years, were assigned to view Medicine 1; all respondents assigned to Susan, age 36 years, were assigned to Medicine 2

^d^289 were assigned; 285 answered these treatment-sequencing questions. Thus all are not included in the sample total

^e^306 were assigned; 302 answered these treatment-sequencing questions. Of the 309 respondents eligible because they were assigned to Medicine 2, 3 did not complete the full survey and are not included in the sample total

The results for the hypothetical woman named Susan varied by Susan’s age. When respondents were told Susan was 25 years old, 55.1% recommended RRBM, 38.6% recommended RRBSO, and 30.5% recommended Medicine 1 as part of Susan’s risk reduction strategy. When Susan was 36 years old, 70.9% recommended mastectomy, 48.7% recommended an oophorectomy, and 22.5% recommended Medicine 2.

**Fig. 2:**
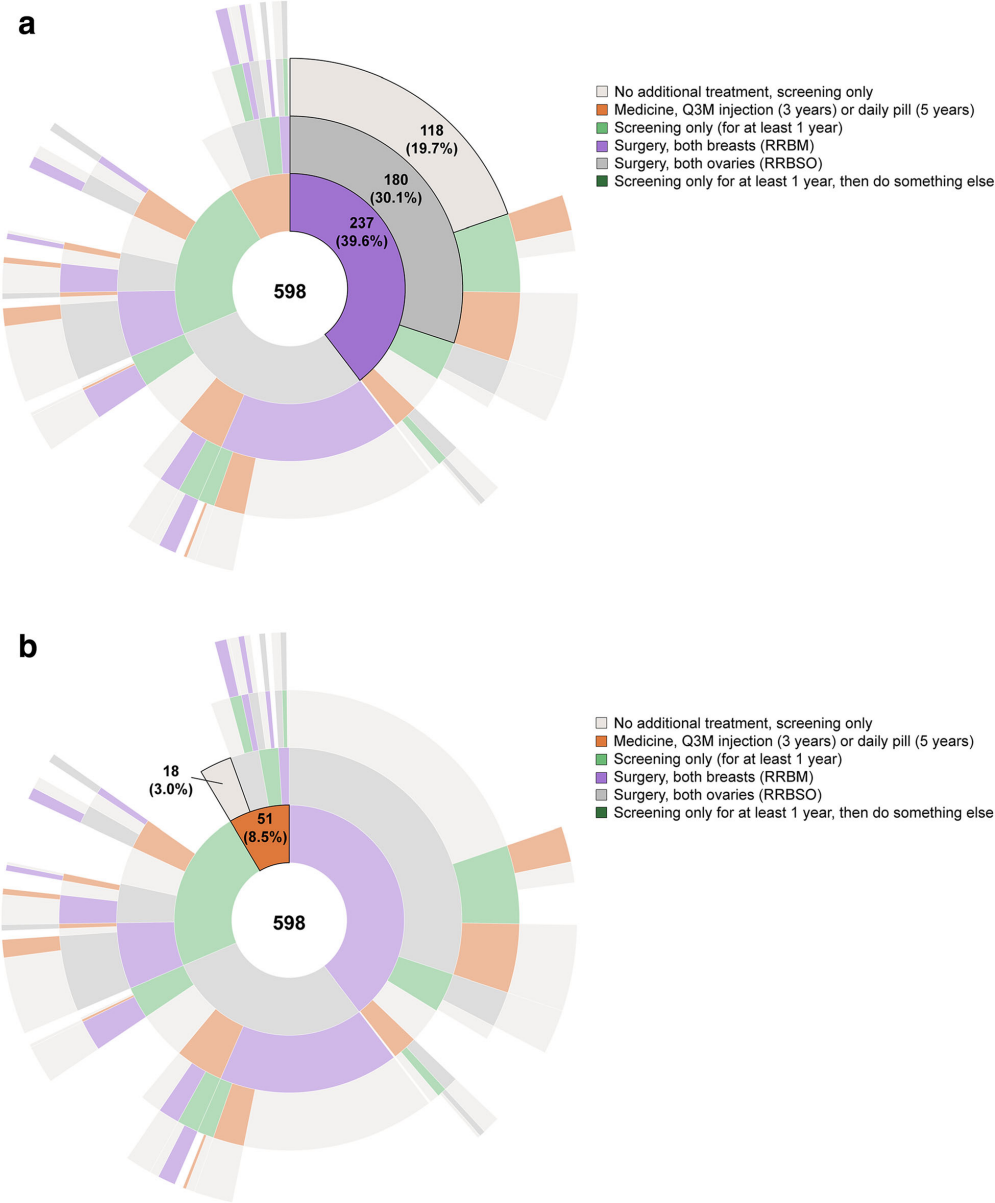
Respondent’s Choices for Herself. a. Path Selected Most Often^a^. b. Path Starting With Medicine Selected Most Often^a^. Q3M = quarterly; RRBM = risk-reducing bilateral mastectomy; RRBSO = bilateral salpingo oophorectomy. ^a^ Sunburst diagram used to visualize hierarchical data using concentric circles. The circle in the center represents the first choice (or root node) as indicated by respondents, with the hierarchy moving outward from the center indicating women’s second, third, and fourth choices. Online, dynamic versions of Sunburst diagrams can be found at https://docs.novisci.com/sunburst+breast+cancer+treatment+survey/

Sunburst diagrams in Fig. 2a and b summarize all the different treatment sequences the respondents selected for themselves (an interactive version of the figure with the percentage of women selecting each treatment sequence is available at https://docs.novisci.com/sunburst+breast+cancer+treatment+survey/). Figure 2a highlights the path selected by the highest percentage of women. The most commonly selected path (19.7% of respondents) started with RRBM, followed by RRBSO, followed by screening only (no additional treatment) (Fig. 2a). This compares to 33% who reported getting both a RRBM and a RRBSO as their initial treatment (Table 1). In addition, the majority of women who had not had RRBM or RRBSO reported that they were very likely or somewhat likely to pursue both surgeries.

While 29.8% of women selected a medicine as part of their treatment sequence, only 8.5% selected a medicine as their initial treatment. Highlighted in Fig. 2b, 3% of women started with a medicine and then no other treatment. This compares with 6% who reported taking a medicine to reduce their risk of breast cancer (Table 1).

**Fig. 3:**
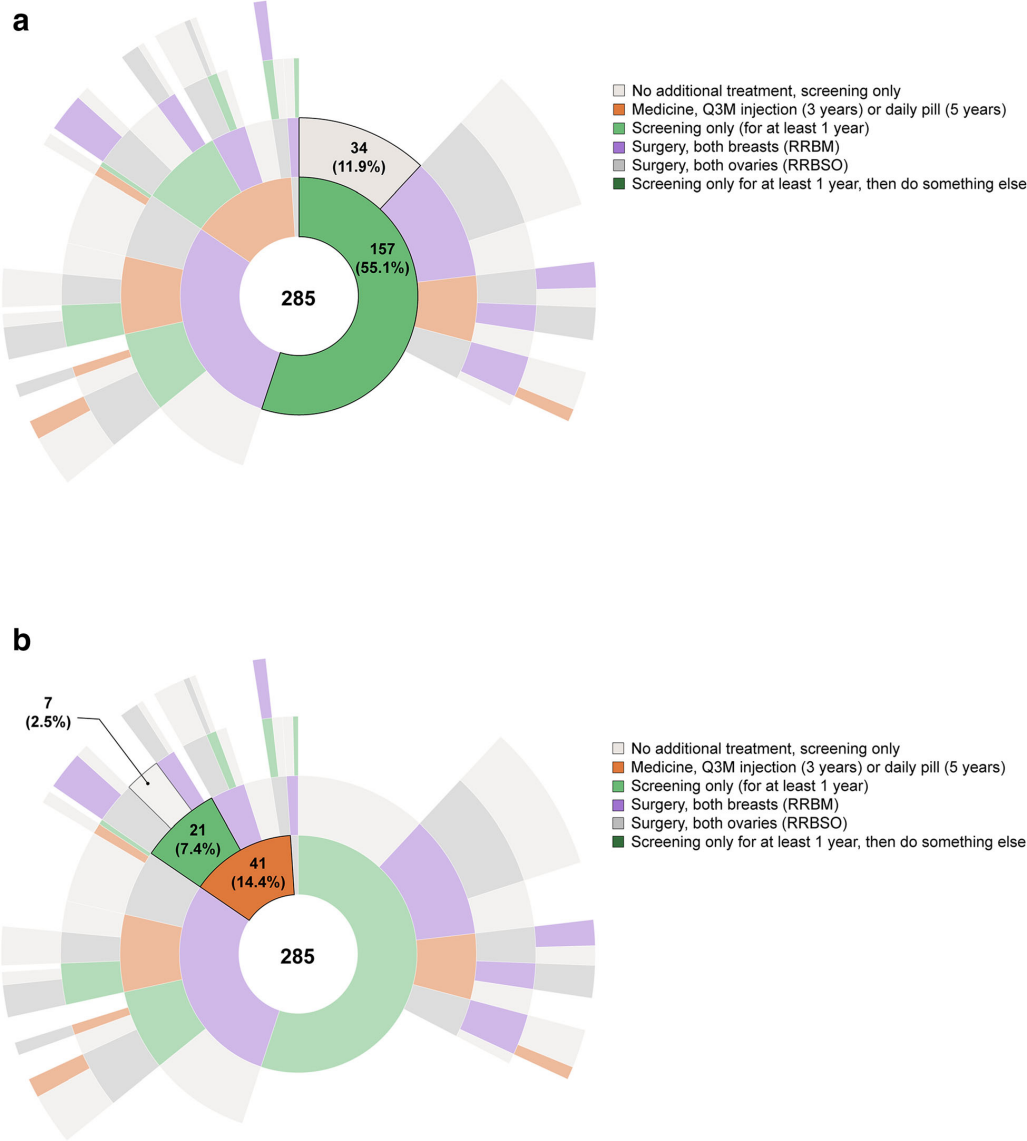
Respondent’s Choices for Susan (25 Years Old). a. Path Selected Most Often^a^. b. Path Starting With Medicine Selected Most Often^a^. Q3M = quarterly; RRBM = risk-reducing bilateral mastectomy; RRBSO = bilateral salpingo oophorectomy. ^a^ Sunburst diagram used to visualize hierarchical data using concentric circles. The circle in the center represents the first choice (or root node) as indicated by respondents, with the hierarchy moving outward from the center indicating women’s second, third, and fourth choices. Online, dynamic versions of Sunburst diagrams can be found at https://docs.novisci.com/sunburst+breast+cancer+treatment+survey/

Figure 3a and b display the different treatment sequences selected for the hypothetical 25-year-old Susan. In Fig. 3a, the most common path selected was screening only (selected by 11.9% of respondents for Susan). A medicine was selected as the first step in treatment for the younger Susan by 14.4% of respondents (Fig. 3b). In Fig. 3b, the most common path that starts with a medicine included the medicine and then screening only (selected by 2.5% of respondents).

**Fig. 4:**
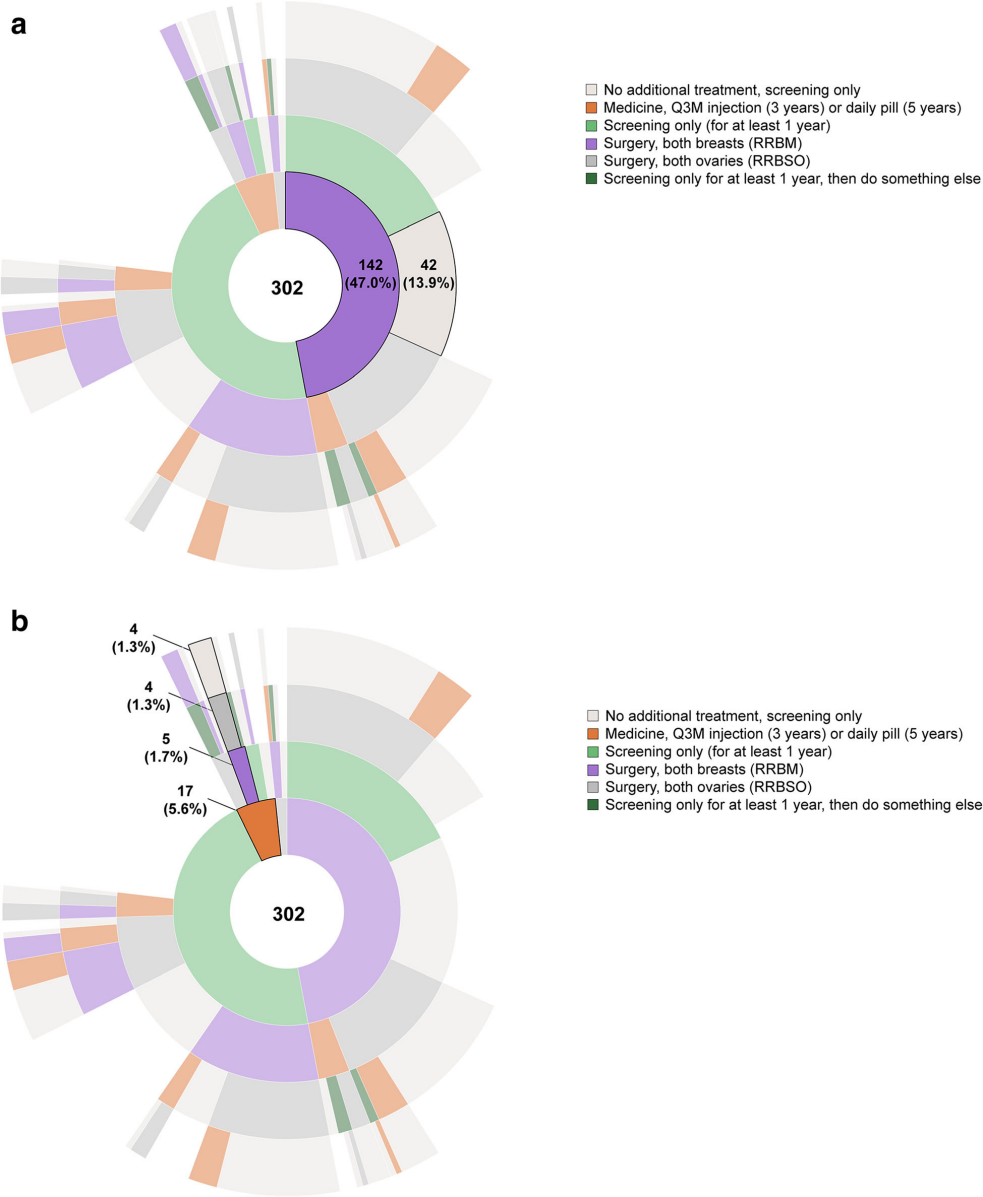
Respondent’s Choices for Susan (36 Years Old). a. Path Selected Most Often^a^. b. Path Starting With Medicine Selected Most Often^a^. Q3M = quarterly; RRBM = risk-reducing bilateral mastectomy; RRBSO = bilateral salpingo oophorectomy. ^a^ Sunburst diagram used to visualize hierarchical data using concentric circles. The circle in the center represents the first choice (or root node) as indicated by respondents, with the hierarchy moving outward from the center indicating women’s second, third, and fourth choices. Online, dynamic versions of Sunburst diagrams can be found at https://docs.novisci.com/sunburst+breast+cancer+treatment+survey/

Figure 4a and b presents the results for the hypothetical 36-year-old Susan. In Fig. 4a, the most common path selected was RRBM followed by screening only (selected by 13.9% of respondents). In Fig. 4b, the most common path starting with a medicine showed RRBM and RRBSO as subsequent treatments (selected by 1.3% of respondents). For the older Susan, 5.6% of respondents recommended Susan start with a medicine. The interactive versions of Figs. 3 and 4 with the full set of results for both hypothetical women are available at https://docs.novisci.com/sunburst+breast+cancer+treatment+survey/.

Table 3 presents the results from the multinomial logit model that examines the characteristics of the women who included a chemoprevention option as part of their hypothetical treatment sequence. In the full sample, the time since learning about their *BRCA1/2* status and prior experience taking a medication (tamoxifen, raloxifene, or an aromatase inhibitor) were associated with a higher likelihood of choosing one of the hypothetical chemoprevention medicines. Respondents with prior experience taking a medication were more than 2.5 times more likely to choose a medication in the treatment sequencing questions than women who had not taken a medication in the past. Women who had both an RRBM and a RRBSO in the past were more than 60% less likely to choose a hypothetical medicine as part of the treatment sequence.

**Table 3:** Logit Models: Predicting Respondents Who Would Choose a Medicine for Herself

Characteristic	All Women (***N*** = 598), OR (95% CI)	Women Assigned to See Medicine 1, a Denosumab-Like Medicine (***n*** = 289), OR (95% CI)	Women Assigned to See Medicine 2, a Tamoxifen-Like Medicine (***n*** = 309), OR (95% CI)
Age 40 years or over	0.953 (0.563–1.614)	1.446 (0.678–3.083)	0.651 (0.298–1.423)
Hopes to have children in future (after mutation identified) or undecided	0.792 (0.468–1.338)	0.772 (0.354–1.683)	0.806 (0.380–1.711)
Time since genetic test	1.051* (1.007–1.097)	1.095* (1.029–1.165)	1.014 (0.952–1.080)
First-degree relative with breast cancer	0.962 (0.657–1.409)	0.821 (0.474–1.422)	1.086 (0.627–1.879)
First-degree relative with ovarian cancer	1.240 (0.795–1.935)	1.401 (0.736–2.669)	1.305 (0.681–2.498)
RRBM only	0.693 (0.398–1.207)	1.284 (0.538–3.063)	0.468* (0.220–0.996)
RRBSO only	0.753 (0.408–1.390)	0.951 (0.385–2.349)	0.546 (0.224–1.331)
RRBM and RRBSO	0.383* (0.211–0.697)	0.331* (0.134–0.816)	0.424* (0.182–0.987)
Higher education	0.843 (0.573–1.241)	0.933 (0.524–1.663)	0.761 (0.440–1.316)
Has taken a prescription medication, such as tamoxifen, raloxifene, or an aromatase inhibitor [anastrazole, exemestane]	2.524* (1.198–5.316)	1.683 (0.565–5.015)	3.506* (1.214–10.124)

*CI* Confidence interval, *OR* Odds ratio, *RRBM* Risk-reducing bilateral mastectomy, *RRBSO* Bilateral salpingo oophorectomy

*Statistically significant at 95% confidence level

Among women assigned to see Medicine 1 (a medicine with characteristics like denosumab), time since genetic testing was associated with a higher likelihood of choosing a medicine and having both an RRBM and a RRBSO was associated with a lower likelihood of choosing a medicine. Among women assigned to see Medicine 2 (a medicine with characteristics like tamoxifen), prior experience taking a medication was associated with a higher likelihood of choosing the hypothetical medicine and having an RRBM and having both an RRBM and a RRBSO were associated with a lower likelihood of choosing a medicine.

In the models predicting the inclusion of a chemoprevention medicine in the recommendations for Susan, respondents were 1.5 times more likely to select a medicine when Susan was 25 than when Susan was 36 (Table 4). Among respondents assigned to 25-year-old Susan and Medicine 1 (a medicine with characteristics like denosumab), none of the variables in the model were associated with a higher likelihood of recommending a medicine. However, among women assigned to 36-year-old Susan and Medicine 2 (a medicine with characteristics like tamoxifen), having a family member (mother, father, sister, brother, daughter, or son) who has been diagnosed with breast cancer and prior experience taking a medication was associated with a higher likelihood of choosing a medicine.

**Table 4:** Logit Models: Predicting Respondents Who Would Choose a Medicine for Susan

Characteristic	All Women (***N*** = 587), OR (95% CI)	Women Assigned to See Susan Age 25 Years and Medicine 1, a Denosumab-Like Medicine (***n*** = 285), OR (95% CI)	Women Assigned to See Susan Age 36 Years and Medicine 2, a Tamoxifen-Like Medicine (***n*** = 302), OR (95% CI)
Age 40 years or over	0.983 (0.568–1.703)	1.342 (0.636–2.831)	0.656 (0.274–1.571)
Hopes to have children in future (after mutation identified) or undecided	1.054 (0.604–1.838)	0.830 (0.378–1.823)	1.347 (0.580–3.129)
Time since genetic test	1.000 (0.957–1.046)	0.989 (0.929–1.052)	1.015 (0.948–1.086)
First-degree relative BC	1.354 (0.898–2.040)	1.072 (0.617–1.864)	1.924* (1.017–3.638)
First-degree relative OC	1.049 (0.659–1.669)	0.946 (0.496–1.807)	1.378 (0.680–2.795)
RRBM only	0.806 (0.441–1.473)	1.30 (0.545–3.392)	0.485 (0.209–1.127)
RRBSO only	1.325 (0.692–2.539)	1.915 (0.748–4.899)	1.010 (0.387–2.637)
RRBM and RRBSO	0.851 (0.456–1.586)	1.227 (0.503–2.993)	0.607 (0.235–1.570)
Higher education	0.880 (0.588–1.316)	1.325 (0.745–2.358)	0.584 (0.321–1.064)
Has taken a prescription medication, such as tamoxifen, raloxifene, or an aromatase inhibitor [anastrazole, exemestane]	1.799 (0.840–3.855)	0.850 (0.265–2.725)	3.321* (1.129–9.771)
Assigned to Susan, age 25 years	1.500* (1.031–2.183)	Not applicable	Not applicable

*BC* Breast cancer, *CI* Confidence interval, *OC* Ovarian cancer, *OR* Odds ratio, *RRBM* Risk-reducing bilateral mastectomy, *RRBSO* Bilateral salpingo oophorectomy

*Statistically significant at 95% confidence level

## Discussion

As new treatment options are developed to reduce the risk of breast and ovarian cancer in women who carry the *BRCA1* or *BRCA2* genetic mutation, it will become increasingly important to understand women’s preferences for risk-reducing treatment. In this study, the largest reported survey of women with a *BRCA1* or *BRCA2* mutation and no personal history of cancer, we described the sequence of treatments according to women’s choices between the current options of RRBM, RRBSO, a medicine like tamoxifen, and a medicine with characteristics like denosumab.

Most of the women in our study selected surgical intervention as part of their cancer-risk reduction treatment sequence—their stated sequence largely mirrored what they had done in their lives. Two-thirds of women in the sample had already had either RRBM or RRBSO, one-third reported having both a RRBM and a RRBSO as their initial treatment. Among those who had not undergone prophylactic surgery at time of survey, most reported that they were still planning to have one or both surgeries in the future—for example, approximately 90% of women planned to have RRBSO in the future. This is not surprising as treatment guidelines, such as the National Comprehensive Cancer Network guidelines [19], recommend RRBSO typically between 35 and 40 years of age and upon completion of childbearing, and 56% of our participants were 40 years of age or older at time of survey. Risk-reducing bilateral salpingectomy with delayed oophorectomy is another surgical option, one that was not evaluated in our study, and which is still under clinical investigation as ovarian cancer prevention such as in the WISP study [20]. Women with prior experience with RRBM and RRBSO were more than 60% less likely to select medication as part of their treatment sequence, and women who had used tamoxifen or another similar medicine were more than 2.5 times more likely to choose a medicine like tamoxifen in the survey. It is possible that respondents recognized the description of tamoxifen from the side effects. Having used tamoxifen in the past, however, was not a predictor of whether women selected the medicine like denosumab in the survey.

The decision to have surgery to reduce the risk of cancer is influenced by a woman’s experience and family circumstances. In our multinational cohort, women with a first-degree relative with a diagnosis of breast cancer were more likely to undergo RRBM and/or RRBSO than women without a first-degree relative with breast cancer (74% versus 54%). In addition, women who have children were more likely to have had RRBM and/or RRBSO than women with no children (76% versus 48%). This finding is supported by studies (and clinical experience) revealing that women without breast cancer who have *BRCA1/2* mutations are more likely to choose RRBM (versus surveillance only) if they have a first- or second-degree relative who died from breast cancer [21], particularly if they have lost a mother or sister at young ages (< 60 and < 50 years, respectively) (D. G. Evans, MD, submitted manuscript). These studies have also reported that number of children a woman has can be predictive of RRBM uptake [21] and D. G. Evens, MD (submitted manuscript).

In our questions about a hypothetical woman, Susan, several differences emerged between recommendations for 25-year old and 36-year old Susan. Overall, the most common path for cancer-risk reduction was RRBM, followed by RRBSO (followed by surveillance, or no intervention) (Fig. 2), although women were less willing to choose surgical interventions for a woman in her 20s and more likely to first recommend RRBM then RRBSO for a woman in her mid-30s. Women were much more likely to recommend that 25-year-old Susan start with a medicine, which may be appropriate based on the absolute risk of breast cancer in this decade of life, even for this population of high-risk women. This pattern was consistent with women’s interest in non-surgical options for risk reduction, especially for those whose *BRCA* mutations are identified at younger ages.

About a third of the respondents indicated they would take a medicine as part of their sequence, which is a much larger proportion than that for those who reported actually taking a medicine. Of these, less than 10% indicated they would use a medicine as their initial therapy. In practice, a medicine would likely only be prescribed before surgery, or less frequently following RRBSO. The observation that many of our respondents stated they would choose a medicine in their treatment sequence suggests an unmet need still exists for additional risk-reducing options. In the discrete-choice experiment among this high-risk population [22], we reported that women highly valued the degree of breast cancer risk reduction and that they desired a safe chemoprevention drug that is currently not available to them.

### Limitations

Our study focused primarily on four English-speaking countries where 93% of the respondents were white/Caucasian of European descent and two-thirds had at least some college-level education; therefore, the results do not provide further insights into choices made by women in other countries with different health care systems, ethnic backgrounds, and socioeconomic circumstances. The survey did not present the marginal risk-reduction of a treatment option conditional on the other actions a woman indicated she would take first, so the survey overstated the reduction in risk for the second, third, and fourth options the women selected. It is possible that women would not have selected some follow-up treatments if they were presented with the lower marginal risk reduction, given the previous treatments selected. In addition, it is possible that if their physician or genetic counselor explained the medication options, more women would have selected them than observed in the survey.

## Conclusions

This study provides insights into the motivations and preferences for risk-reducing strategies in a large international sample of unaffected women with a *BRCA1* or *BRCA2* mutation. The responses reveal a willingness by women to undergo risk-reducing surgeries, both RRBM and RRBSO, which afford the largest reduction in breast cancer risk. However, the women were less willing to choose surgical interventions for a woman in her 20s and supported the use of a chemoprevention option for a 25-year-old woman as a reasonable strategy before elective surgeries. Surgical intervention with RRBM and RRBSO, in this order, would be recommended for a woman who is 36 years of age. Ultimately, achieving the highest possible degree of breast cancer risk reduction is the most important consideration of these high-risk women, regardless of whether it is non-surgical or surgical intervention, and many more women would take a chemoprevention agent compared to how many actually had taken a drug to prevent disease. These findings suggest that the uptake of chemoprevention by women with a *BRCA* mutation may be higher, given the availability of a safe and effective drug.

## Data Availability

The data that support the findings of this study are available from the corresponding author upon reasonable request.

## Abbreviations

BC: Breast cancer
CI: Confidence interval
FORCE: Facing Our Risk of Cancer Empowered
ONJ: Osteonecrosis of the jaw
OC: Ovarian cancer
OR: Odds ratio
Q3M: Quarterly
RANKL: Receptor activated nuclear factor kappa-B ligand
RRBM: Risk-reducing bilateral mastectomy
RRBSO: Risk-reducing bilateral salpingo oophorectomy
SD: Standard deviation
